# SARS-CoV-2 Receptor Binding Domain (RBD) Protein–Protein Conjugate Induces Similar or Better Antibody Responses as Spike mRNA in Rhesus Macaques

**DOI:** 10.3390/vaccines13060648

**Published:** 2025-06-17

**Authors:** Puthupparampil V. Scaria, Christopher G. Rowe, Ivan Kosik, Zhe Hu, Jonathan P. Renn, Nada Alani, Pinar Kemanli, Sachy Orr-Gonzalez, Lynn E. Lambert, Kayode Adeyemi, Justin Y. A. Doritchamou, Emma K. Barnafo, Kelly M. Rausch, Liya Muslinkina, Robert D. Morrison, John-Paul Todd, Dominic Esposito, Andrew Lees, Jonathan Yewdell, Patrick E. Duffy

**Affiliations:** 1 Laboratory of Malaria Immunology and Vaccinology, National Institute of Allergy and Infectious Diseases, National Institutes of Health, 29 Lincoln Drive, Building 29B, Bethesda, MD 20892, USA; puthupparampil.scaria@nih.gov (P.V.S.); rowechris@niaid.nih.gov (C.G.R.); jonathan.renn@nih.gov (J.P.R.); nada.alani@nih.gov (N.A.); pinar.kemanli@nih.gov (P.K.); satos@niaid.nih.gov (S.O.-G.); lelambert@niaid.nih.gov (L.E.L.); kayode.adeyemi@nih.gov (K.A.); yai.doritchamou@nih.gov (J.Y.A.D.); barnafoe@niaid.nih.gov (E.K.B.); krausch@niaid.nih.gov (K.M.R.); robert.morrison@nih.gov (R.D.M.); 2 Laboratory of Viral Diseases, National Institute of Allergy and Infectious Diseases, National Institutes of Health, Bethesda, MD 20892, USA; ivan.kosik@nih.gov (I.K.); zhe.hu@nih.gov (Z.H.); jyewdell@niaid.nih.gov (J.Y.); 3 Structural Biology Section, Research Technologies Branch, National Institute of Allergy and Infectious Diseases, National Institutes of Health, Bethesda, MD 20892, USA; liya.muslinkina@nih.gov; 4 Vaccine Research Center, National Institute of Allergy and Infectious Diseases, National Institutes of Health, Bethesda, MD 20892, USA; jptodd@mail.nih.gov; 5 Protein Expression Laboratory, Cancer Research Technology Program, Frederick National Laboratory for Cancer Research, Frederick, MD 21701, USA; dom.esposito@nih.gov; 6 Fina BioSolutions LLC, 9430 Key West Ave. Suite 200, Rockville, MD 20850, USA; alees@finabio.com

**Keywords:** SARS-CoV-2, receptor binding domain, protein–protein conjugate, rhesus

## Abstract

**Background/Objectives**: Rapid development of vaccines against SARS-CoV-2 was pivotal to controlling the COVID-19 pandemic. The emergency also provided a rare opportunity to test novel vaccine platforms such as mRNA in large clinical trials. Most of the early vaccines used SARS-CoV-2 Spike protein as the target antigen. Nevertheless, subsequent studies have shown that Receptor Binding Domain (RBD) of Spike also can yield efficacious vaccines, and we previously demonstrated that chemical conjugation of RBD to a carrier protein, EcoCRM^®^, enhanced antibody responses and induced strong virus neutralization activity in mice. **Methods**: Here, we compared the immunogenicity of this conjugate to that of an approved mRNA vaccine from Pfizer/BioNTech in rhesus macaques over a period of nine months. **Results**: AS01-adjuvanted RBD conjugate induced a similar or better antibody response, receptor binding inhibition, and virus neutralization activity against different variants of SARS-CoV-2, compared to mRNA. IgG subclass profiles induced by conjugate and mRNA vaccines were initially dominated by IgG1 and IgG3 then switched to IgG2 and IgG4 dominant profiles during the subsequent six-month period. Polyclonal immune sera from the conjugate and mRNA had similar antibody avidity at multiple time points. **Conclusions**: In summary, antibody responses in rhesus macaques induced by the RBD-EcoCRM conjugate and the Spike mRNA vaccine are very similar. These results demonstrate the potential for the RBD-EcoCRM conjugate as a vaccine against SARS-CoV-2.

## 1. Introduction

The COVID-19 pandemic led to numerous vaccine development efforts using a variety of well-established as well as novel vaccine technologies [1,2,3,4,5,6]. The urgent need and unprecedented investments provided a rare opportunity to rapidly test novel vaccine technologies such as mRNA in large clinical trials and demonstrate their safety and effectiveness. These world-wide efforts resulted in rapid development of several effective vaccines against SARS-CoV-2 that were pivotal to controlling the pandemic [7]. Despite these successes, many low- and middle-income countries (LMICs) did not gain access to highly efficacious vaccines until late in the emergency. The development of accessible and cost-effective vaccines is a clear priority for LMICs to combat both endemic and pandemic diseases.

In the USA, two mRNA vaccines, COMIRNATY^®^ and Spikevax^®^, progressed rapidly to licensure, followed later by the viral vector-based vaccine Janssen COVID-19 vaccine (from Johnson & Johnson, Brunswick, NJ, USA) which had safety concerns and limited availability [8]. The only protein-based vaccine that gained emergency use authorization, Novavax COVID-19 Vaccine, Adjuvanted, entered the USA market in July 2022. Countries outside the USA utilized a mix of inactivated virus, protein subunit, and adeno-vector-based vaccines, which were also developed relatively fast, to address the emergency [4,6,9,10]. While most of the vaccines developed against the parent strain of the virus were highly effective, their efficacy decreased as the virus evolved, necessitating the development of updated vaccines against the newer variants [11,12,13]. mRNA/LNP has proven to be an effective COVID vaccine platform; however, an elevated incidence of myocarditis, especially in younger adult male vaccinees, is a safety issue that warrants further evaluation [14,15,16].

Chemical conjugation of polysaccharide antigens to protein carriers has been a successful approach to develop safe, effective, and relatively low-cost anti-microbial conjugate vaccines [17,18]. Chemical conjugation to a protein carrier enhances polysaccharide immunogenicity by transforming a T-independent to a T-dependent immune response. Similarly, protein–protein conjugation, wherein a poorly immunogenic protein antigen is chemically conjugated to a carrier protein, has also proven to be an effective vaccine delivery platform [19,20,21]. For example, conjugation of malaria transmission-blocking antigens such as Pfs25 and Pfs230D1 to carrier proteins like Exoprotein-A, CRM197, and Tetanus Toxoid has enhanced their immunogenicity, and some of these conjugates have advanced in clinical development ([22,23,24], Clinical Trial IDs: NCT05135273, NCT03917654). This approach is especially suitable for smaller protein subunit antigens that are not highly immunogenic.

We adapted the protein–protein conjugate vaccine technology to develop a COVID vaccine candidate by chemically conjugating the Receptor Binding Domain (RBD) of SARS-CoV-2 Spike protein to the carrier protein EcoCRM^®^ produced in *E. coli*, a recombinant biosimilar of CRM197 [25,26]. Although initial COVID vaccines used full-length Spike protein as the antigen, vaccines based on the receptor binding domain (RBD) of Spike also have been highly effective [27]. RBD-based vaccines focus the immune response on the region involved in the binding of the virus to ACE2 receptors on the human cell surface. The majority of neutralizing antibodies induced by Spike immunogens target RBD [28,29]. Thus, vaccines using RBD antigen can reduce the number of non-neutralizing antibodies generated, benefiting the development of updated booster vaccines needed to respond to virus evolution.

RBD vaccine candidates using several different platforms have demonstrated their effectiveness in preclinical studies [27,30,31,32]. RBD-based vaccines have also been efficacious in clinical trials and approved for human use in Cuba, India, and other countries [33,34,35]. We previously described two different types of protein–protein conjugates based on RBD antigen, and we tested these in mouse studies [25]. One of our RBD candidates had multiple antigen and carrier molecules chemically crosslinked to form large-molecular-weight conjugates with an average molecular weight of 1364 kDa, referred to as “cross-linked conjugate”. In the second case, antigens were tethered to the carrier without inducing crosslinking between them, leading to conjugates with an average molecular weight of 220 kDa, referred to as “radial conjugate”. While both types of conjugates were highly immunogenic compared to unconjugated antigen in mouse studies, only the radial conjugate induced a robust neutralizing antibody response against the virus.

Here, we evaluated the RBD radial conjugate vaccine in rhesus macaques at two different doses (40 and 20 µg per dose) and formulated in the GSK adjuvant AS01, which is used in the licensed Mosquirix™ and SHINGRIX vaccines against malaria and shingles, respectively. The study compared the RBD radial conjugate vaccine to an mRNA vaccine encoding full-length Spike, thus providing a direct comparison between the immune response induced by RBD and Spike antigens, although using two different vaccine platforms. Conjugates induced high levels of antibody against RBD, as well as full length Spike, similar or higher to those induced by mRNA. In ACE2 receptor binding inhibition and virus neutralization assays, conjugates showed very similar or higher activities as the mRNA vaccine against various strains of SARS-CoV-2. Both vaccines induced a durable immune response against the WA1 virus, but the virus neutralization activity was lower against Omicron variants. IgG subclass analysis of immune sera showed similar antibody profiles in radial conjugate versus mRNA vaccines. We conclude that protein–protein conjugation is a feasible and attractive technology for vaccines against SARS-CoV-2 and potentially other infectious diseases.

## 2. Materials and Methods

### 2.1. Animals

Animal studies were performed at NIAID in accordance with the animal protocol approved by the NIAID Animal Care and Use Committee (ACUC; Approval Code LMIV 3; Approved on 1 June 2022). All procedures were carried out as per the Guide for the Care and Use of Laboratory Animal Reports NIH 85-23. Twenty-four healthy, juvenile, naïve rhesus macaques (*Macaca mulatta*) were randomized by age, sex, and weight into three groups of eight animals per group (the sample size was based on a power calculation to assess the significance of a 2-fold difference in the vaccine-induced antibody titer). Macaques were socially housed and maintained in an AAALAC-accredited NIAID facility (file #000777, last accredited in 2024). Two groups of animals were vaccinated on days 0, 28, and 112, with RBD-EcoCRM conjugate at a 40 µg (Conj-40) or 20 µg (Conj-20) antigen (RBD) dose, in a volume of 0.5 mL per vaccination. The 40 µg dose corresponds to the dose used in the human trials of a similar protein–protein conjugate vaccine against malaria and found to be safe [23]). A third group of animals were vaccinated on days 0, 28, and 112 with 0.3 mL containing Pfizer/BioNTech mRNA/LNP (30 µg mRNA per vaccination). Vaccines were administered via intramuscular injection in the leg, alternating legs between successive injections. Animals were physically examined on days 1, 2, 3, 7, and 14 post-immunizations and monitored daily for general health and well-being, in consultation with a certified veterinarian in case of pain, distress, or soreness. Vaccines were well tolerated, and no adverse effects, local or systemic, were observed in any of the animals during the study. The study was not blinded; all technicians and researchers were aware of the group allocation throughout the experiment.

Animals were bled on days 0, 7, 14, 28, 35, 42, 56, 84, 112, 119, 126, 140, 168, 196, 224, 252, and 280. Sera were analyzed for the antibody response against RBD and full-length Spike from the WA1 variant. Sera from day 42 (2 weeks post vac 2), day 126 (2 weeks post vac 3), and day 280 (24 weeks post vac 3) were analyzed for ACE2 receptor binding inhibition activity (BIA) and virus neutralization (VN) activity against variants of SARS-CoV-2. The IgG subclass distribution and avidity of the polyclonal sera of conjugate groups were compared to the mRNA group at multiple time points post vaccination. No animals, experimental units, nor data points were excluded in the analysis.

### 2.2. RBD Conjugate

The Receptor Binding Domain (RBD) antigen used in this study corresponds to amino acids AA319 through AA541 of wild-type SARS-CoV-2 (WA-1) Spike protein. This protein antigen, expressed in Expi293 cells (Thermo Fisher, Waltham, MA, USA; CAT A14527), has an unpaired Cysteine (Cys) near the carboxyl end that was used to conjugate the antigen to a carrier protein, EcoCRM^®^, through thioether chemistry. EcoCRM^®^, a recombinant protein carrier with a molecular weight of 58,412.6 Da, was produced in *E. coli* (Fina Biosolutions LLC, Rockville, MD, USA). Synthesis and characterization of the conjugate has been described in detail previously, and the RBD-EcoCRM conjugate used in this study has a molecular weight of 299 kDa by Size Exclusion Chromatography with Multi-Angle Light Scattering (SEC-MALS) and antigen content of 70.8% (*w*/*w*) or a molar ratio of 5.7 (antigen/carrier) by amino acid analysis discussed previously [25].

### 2.3. mRNA

mRNA/LNP used in this study was collected from unused portions of Pfizer/BioNTech vaccine vials (Lot# EW0170) that were opened for human vaccination: unopened vials of mRNA vaccines were not available for any use other than human vaccination at the time of this study. Residual vaccine collected from opened vials was pooled, refrozen, and stored at −80 °C until thawed 4 h prior to rhesus vaccination.

### 2.4. Adjuvant

AS01 [TLR4 agonist MPL (100 µg/mL) and saponin fraction QS-21 (100 µg/mL) in a liposomal formulation] was obtained from the commercially available SHINGRIX^®^ vaccine package. RBD conjugate in 4 mM PBS at pH 7.2 was mixed with an equal volume of adjuvant to prepare the formulation for administration. Formulations were kept at 2–8 °C and administered within 4 h of preparation. The final delivery dose contained 25 µg/25 µg (MPL/QS-21) per 0.5 mL dose. mRNA/LNP was thawed from −80 °C and kept at 2–8 °C until vaccine administration. The mRNA vaccine was administered within 4 h of thawing from −80 °C. Separate syringes were used for each animal vaccination.

### 2.5. IgG and IgG Subclass ELISA Analyses

ELISA was carried out as described previously [25]. Flat-bottom microtiter ELISA plates (Immulon 4 HBX) from Dynex Technologies (Chantilly, VA, USA) were coated with 1 μg/mL of RBD or full-length Spike ectodomain protein from SARS-CoV-2 WA1 in a volume of 100 μL per well in a carbonate coating buffer (pH 9.6) overnight at 4 °C. The next day, wells were blocked for 2 h with 5% skim milk in TBS blocking buffer in a volume of 320 μL per well. Samples were serially diluted in TBS/5% milk and added to wells in triplicate in a volume of 100 μL per well, followed by incubation at room temperature for 2 h. Plates were washed 4 times and alkaline phosphatase labeled goat anti-monkey secondary antibody (Seracare Life Sciences, Milford, MA, USA) diluted at 1:2400 was added in a volume of 100 μL per well and incubated at room temperature for 2 h. After washing four times, 100 μL of phosphatase substrate (dissolved tablets, Sigma, St. Louis, MO, USA) were added to each well, incubated for 20 min and optical densities (ODs) at 450 nm were measured using a SpectraMax M3 microplate reader (Molecular Devices, San Jose, CA, USA). Each ELISA plate contained an internal serum standard from which a four-parameter curve was calculated with SoftMax Pro (version 7.1.2) software. The OD values were converted to ELISA Units using the standard curve.

IgG subclass analyses were performed as follows. Immulon 4 HBX flat bottom ultra-high binding polystyrene microtiter ELISA plates (Thermo-Fisher Scientific, Ref#3855) were coated with 100 ng of wild-type variant of SARS-CoV-2 Spike protein in a carbonate coating buffer (pH 9.6), and incubated overnight at 4 °C. After blocking the plates with 5% milk in Tris-buffered saline (TBS) (blocking buffer) for 1 h at room temperature (RT), samples were diluted at 1:50 in the blocking buffer and added in duplicated wells for 1 h incubation at RT. Plates were washed 4 times with PBS supplemented with 0.05% Tween 20 (PBS-T) and blotted dry. For IgG1, IgG2, and IgG4, secondary mouse antibody specific to each IgG subclass (Anti-rhesus IgG1 [ena], Nonhuman Primate Reagent Resource (NHPRR) Ref#PR-7110, RRID:AB_2819310 at 1:5000 dilution; Anti-rhesus IgG2 [dio], NHPRR Ref#PR-0002, RRID:AB_2895607 at 1:1000 dilution; Anti-rhesus IgG4 [tessera] NHPRR Ref#PR-7186, RRID: AB_2819323 at 1:1000 dilution) was added to the corresponding well/plate and incubated 1 h at RT. Plates were washed, and a tertiary Goat anti-mouse IgG conjugated to HRP (Southern Biotech, Birmingham, AL, USA Ref#1070-05) at 1:10,000 dilution was added to the plates. For IgG1/3 and IgM, HRP-conjugated Goat anti-rhesus IgG1/3 [1B3] (NHPRR Ref#PR-1234, RRID: AB_2819289) and Goat anti-human IgM (Jackson Immuno Research West Grove, PA, USA Ref#109-035-011) were used at 1:1000 dilution for 1 h incubation at RT. After washing the plates, 100 μL of 1-Step TMB ELISA Substrate Solutions (Thermo Scientific Ref#34029) was added to the plates and incubated for 8 min before stopping the reaction with 100 μL of ELISA Stop Solution (Invitrogen, Waltham, MA, USA, Ref# SS04). The OD was measured at 450 nm with a SpectraMax M3 (Molecular Devices) microplate reader.

### 2.6. Receptor Binding Inhibition

The serum antibody inhibition of Spike protein binding to the ACE2 receptor was evaluated as follows, based on a previously published protocol with some modifications [36]. Briefly, Immulon 4 HBX 96-well plates were coated with 10 ng/well of WA1 and Beta variants of SARS-CoV-2 full-length ectodomain spike protein containing 2 stabilizing proline mutations and a mutated furin cleavage site. The protein was diluted in 50 mM Sodium-Carbonate buffer (pH:9.5) and incubated overnight at 4 °C. Plates were washed 3 times with 200 µL PBST and then blocked with 200 µL Phosphate-buffered saline +0.01% *v*/*v* Tween 20 (PBST) +2% *w*/*v* Bovine serum albumin (BSA) at room temperature (RT) for 1 h. After blocking, plates were washed 3 times, and sera diluted at 1:10^2^, 1:10^3^, 1:10^4^, and 1:10^5^ in the blocking buffer were added to the plates (100 µL/well), followed by 20 µL of 60 nM of biotinylated ACE2-Fc at a 10 nM final concentration. A pool of naïve rhesus sera (Day 14) was used as the assay control. After 1 h incubation at RT, plates were washed 3 times, and 200 µL of 100 ng/mL Streptavidin-HRP (ThermoFisher, Cat#21124) was added to wells, then incubated at RT for 1 h. Plates were washed, and 70 µL of 3,3′,5,5′-Tetramethylbenzidine (TMB, SIGMA-Aldrich) was added to the wells and incubated in the dark for 20 min at RT. The substrate reaction was deactivated by adding 70 µL of 0.16 M H_2_SO_4_ solution. Absorbance was measured at 450 nm using a SpectraMax M3 microplate reader (Molecular Devices) and reported as OD. Wells with ‘No ACE2’ (Blank) were used as background controls, while those with ‘No serum’ were used to determine the maximum signal of the ACE2 receptor binding to the antigens. Each study sample was run in duplicate, and triplicated wells of ‘No ACE2’ and ‘No serum’ controls were used in each plate. The average OD of ‘No ACE2’ wells was subtracted from the sample’s OD (Background subtraction) and divided by the average OD of ‘No serum’ wells. The percent inhibition of Spike/ACE2 binding at different serum dilutions was calculated as %Inhibition = 100 × (1 − Background subtracted OD/Average ‘No serum’ OD). ID_50_, the serum dilution at which 50% inhibition occurs, for each animal sera was determined using a 4-parameter fitting with GraphPad Prism software Version 10.

### 2.7. Virus Neutralization Assay

Virus neutralization assays were carried out using replication-competent rOC43-CoV2s, consisting of Spike from different variants and expressing eGFP. rOC43-CoV2s were generated as described earlier [37]. The virus neutralization activity of immune sera from days 42, 126, and 280 for each animal was assayed using rOC43-CoV2 expressing spike proteins from WA1, Delta, Om/B.1.1.529, Om/BA2.86, and Om/EG.5.1 as follows. Sera at different dilutions were incubated with virus for 1 h at 37 °C. Antibody-virus mixtures were added to hACE2-expressing BHK-21 cell monolayers (cell line production described in [38]) in 96-well plates and incubated at 35 °C for 18 h. Subsequently, cells were overlaid with Gibco Minimum Essential Media (MEM) supplemented with 2% FBS. Single-cell suspensions were harvested 18 h later by treating them with TrypLE (Gibco, Waltham, MA, USA) and fixing them with 4% paraformaldehyde (PFA) in PBS for 20 min at room temperature. Cells were washed thrice and resuspended in PBS supplemented with 0.1% BSA. The fraction of cells expressing eGFP (a proxy for viral infection) was measured by flow cytometry (BD Celesta, San Jose, CA, USA). Analysis was performed using FlowJo software Version 10.10.0 (TreeStar). We normalized the frequencies of GFP-positive cells by non-Ab-treated samples and fitted nonlinear regression curves using the dose–response inhibition model for the neutralization assay or one-site binding for the epitope access assay with GraphPad Prism software. Virus infections were performed in the NIAID SARS-CoV-2 Virology Core BSL-3 laboratory.

### 2.8. The Dissociation Rate Constant, k_off_

The rate of dissociation of the RBD/antibody complex was estimated by biolayer interferometry (BLI) using a Sartorius Octet R8 instrument [39]. All measurements were carried out in 10× KB buffer (10× PBS pH7.4, 0.1% BSA, and 0.02% Tween 20). Sensors were incubated for 60 s in 10× KB buffer to establish a baseline prior to loading with recombinant Hisx6-RBD at 1.25 µg/mL in 10× KB buffer for 3 min. Loaded sensors were then equilibrated with 10× KB buffer for 180 s, after which they were exposed to 500-fold diluted NHP serum samples for 180 s. Finally, the sensors exposed to diluted NHP serum were placed in 10× KB to observe the dissociation of the captured antibodies. Ni-NTA sensors loaded with 1.25 mg/mL control antigen Hisx6-Pvs230D1 were the reference sensors for pairwise correction of non-specific interactions between the Ni-NTA sensor surface and NHP serum. Data analysis was carried out using Octet^®^ Analysis Studio software 12.2.0.20, with a Savitzky–Golay filter and a 1:1 fit model, to determine the k_off_ (s-1) values for each measurement. The final values represent an average of three measurements for each time point.

### 2.9. Data Analysis

General Estimating Equations (GEE) analysis using R version 4.4.3 was used to determine the statistical difference in RBD and Spike ELISA units of Conj-40 and Conj-20 compared to the mRNA group from the repeated measurement data at different time points (from day 14 to day 280) during the study. Log-transformed ELISA titers were modeled against all the vaccine groups using an exchangeable correlation structure. Statistical differences between different groups (*p* ≤ 0.05) at specific time points were measured using a Kruskal–Wallis analysis, followed by a Dunn’s post hoc test for pairwise comparisons when appropriate, using Prism v10.0 by GraphPad Software, Inc. ID_50_ values for receptor binding inhibition and virus neutralization were determined using a 4-parameter curve fit of the %inhibition vs. serum dilution for sera at specified time points. Spearman correlation analyses were carried out in Prism to determine correlation coefficients and statistical significance.

## 3. Results

### 3.1. RBD Conjugate Induces Strong Immune Responses Against RBD and Full-Length Spike Protein

RBD-EcoCRM radial conjugate was synthesized as described earlier (25). The RBD antigen corresponding to AA319 through AA541 of the WA1 (WT) Spike protein was conjugated to the EcoCRM^®^ carrier through the unpaired Cys side chain using thioether coupling chemistry (Supplementary Figure S1). The RBD-EcoCRM conjugate had an average molecular weight of 299 kDa, by SEC-MALS analysis, and an average of 5.7 molecules of RBD conjugated to a molecule of EcoCRM^®^, as determined by amino acid analysis.

**Figure 1:**
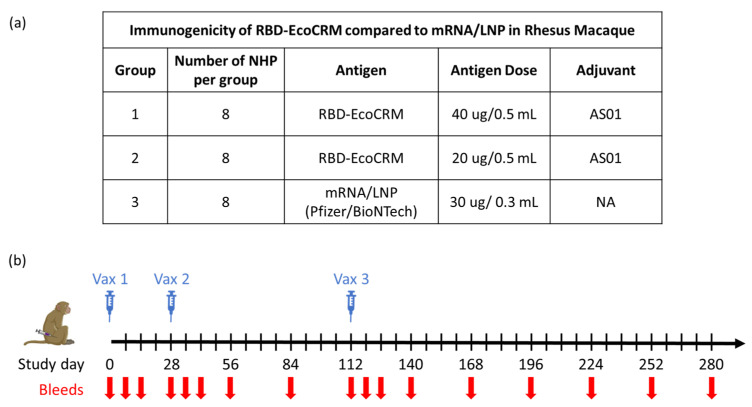
Study plan to evaluate the immunogenicity of RBD-EcoCRM conjugate in rhesus macaques with details of different test groups, dose, group size, and adjuvant used for vaccination study (**a**) and schematic showing vaccination and bleed schedule (**b**).

The immunogenicity of the conjugate with AS01 adjuvant and the mRNA/LNP vaccine were evaluated in rhesus macaques (Figure 1a,b). Three groups of animals (*n* = 8 per group) were vaccinated on days 0, 28, and 112 with either of two different doses, 40 µg and 20 µg (referred to subsequently as Conj-40 and Conj-20, respectively) of RBD conjugate or the human dose (30 µg) of mRNA/LNP vaccine. Animals were bled at different time points through day 280 (24 weeks post Vac. 3) for analyses of the serum antibody response and functional activity.

**Figure 2:**
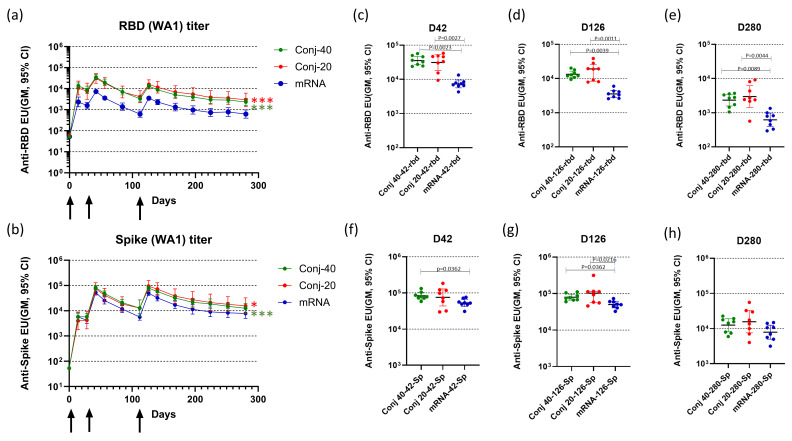
**Vaccination induced strong serum antibody levels against RBD and full-length Spike protein**: Three groups of eight rhesus monkeys were vaccinated with RBD-EcoCRM conjugate at two different doses (40 and 20 µg per dose/animal) or mRNA/LNP (30 µg mRNA per dose per animal) on days 0, 28, and 112. Conjugates, at the indicated dose adjuvanted with AS01, were administered by IM injection of 0.5 mL formulation. In addition, 0.3 mL of mRNA/LNP was administered by IM injection. Serum antibody levels were assayed against RBD (**a**) and full-length Spike (**b**) until day 280. Sera from each animal were analyzed by ELISA in triplicate, and the y-axis represents the geometric mean of ELISA units with a 95% confidence interval. Black arrows indicate days of vaccination. Differences between Conj-40 and Conj-20 vs. mRNA were analyzed by GEE analysis; red asterisks indicate a significant difference between Conj-20 vs. mRNA, while green asterisks indicate a significant difference between Conj-40 vs. mRNA; * *p* < 0.05, *** *p* < 0.001. (**c**–**h**) show the antibody levels against wild-type RBD and full-length Spike, respectively, on days 42 (2 weeks post vaccination 2), 126 (2 weeks post vaccination 3), and 280 (24 weeks post vaccination 3). Error bars represent the geometric mean with a 95% CI. GEE analysis of data from repeated measurements from day 14 to day 280 showed significantly higher ELISA units for conjugates compared to mRNA against RBD ((**a**) *p* < 0.001 for mRNA vs. Conj-40 and Conj-20) and against Spike ((**b**) *p* < 0.001 for mRNA vs. Conj-40 and *p* = 0.04 for mRNA vs. Conj-20). Statistical differences between groups on days 42, 126, and 280 were measured using a Kruskal–Wallis one-way ANOVA followed by a Dunn’s multiple comparison test.

Figure 2a,b show the serum antibody levels against WA1 RBD and full-length Spike protein, respectively, at various time points. The first vaccination induced a strong immune response against RBD, as well as full-length Spike, based on the antibody response measured on day 14, two weeks post vaccination 1 (Figure 2a,b). All animals in the two conjugate groups, as well as the mRNA group, seroconverted after the first vaccination. Antibody responses were further boosted by the second vaccination on day 28, with a 4.0-, 4.1-, and 4.6-fold increase against RBD and a 14.5-, 17.6-, and 12.5-fold increase against full-length Spike, respectively, for the Conj-40, Conj-20, and mRNA groups. A third vaccination on day 112 did not result in increases in antibody levels above the peak levels observed after the second vaccination. Serum antibody levels against RBD were similar for the two conjugate groups but were significantly higher than the mRNA group throughout the period of study. Antibody levels of Conj-40 and Conj-20 were significantly higher than that from mRNA during the course of the study by GEE analysis of repeated measurements (Figure 2a) (*p* = <0.001 for mRNA vs. Conj-40 and Conj-20), as well as analyses of the three groups at specific time points by Kruskal–Wallis analysis (Figure 2c–e). Even though the geometric mean titers of the two conjugate groups Conj-40 and Conj-20 were not significantly different, the Conj-20 group showed higher variability among the animals within the group. Antibody levels against Spike protein were higher than that against RBD for the mRNA group, as expected, since mRNA codes for full-length Spike. Surprisingly, levels of antibody against Spike were higher compared to RBD for the conjugate groups, as well. Though the difference between the Spike antibody levels in the conjugate and mRNA groups was less pronounced, the conjugate groups still had higher antibody titers compared to the mRNA group after the second vaccination. Anti-Spike antibody titers were significantly higher for both conjugates compared to mRNA by GEE analysis (*p* < 0.001 for mRNA vs. Conj-40 and *p* = 0.04 for mRNA vs. Conj-20) (Figure 2b). On day 42 and day 126, sera from Conj-40 and Conj-20 produced significantly higher antibody levels compared to mRNA, whereas the differences were not significant for day 280 sera (Figure 2f–h).

The third vaccination did not increase peak-titers above those seen post dose-2; however, antibody decay was more prolonged versus post dose 2 for all three groups. Geometric mean titers for the Conj-40, Conj-20, and mRNA groups against RBD decreased by 10.7-, 7.45-, and 11.8-fold, respectively, during the 70-day period (from day 42 to day 112) after the second vaccination, whereas the decrease during the 70-day period (day 126 to day 196) after the third vaccination was 3.3-, 2.9-, and 3.6-fold, respectively. A similar trend was observed for the antibody titer against full-length Spike. While geometric mean titers decreased by 8.0-, 5.9-, and 9.3-fold from day 42 to day 112, the corresponding decrease from day 126 to day 196 was 3.6-, 3.4-, and 4.4-fold, respectively, for the Conj-40, Conj-20, and mRNA groups. By day 280, 24 weeks post vaccination 3, antibody levels decreased by 5.7-, 5.4-, and 5.7-fold, respectively, for the Conj-40, Conj-20, and mRNA groups against RBD and 6.3-, 6.0-, and 6.3-fold, respectively, against Spike, from the peak titers on day 126.

### 3.2. Spike-ACE2 Binding Inhibition Activity of Immune Sera

**Figure 3:**
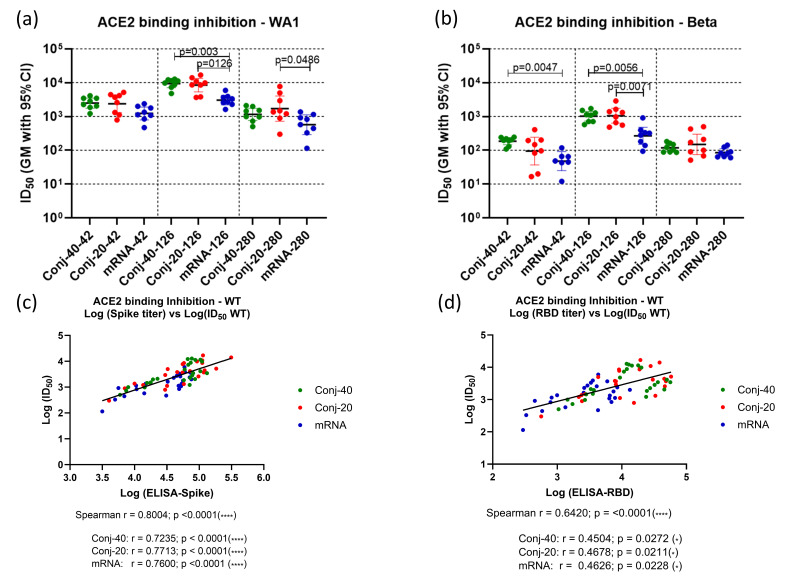
**Immune sera show strong receptor binding inhibition activity:** Immune sera collected on days 42 (2 weeks post vac 2), 126 (2 weeks post vac 3), and 280 (24 weeks post vac 3) from each animal vaccinated with RBD conjugate or mRNA were assessed by ELISA and ID_50_, defined as the dilution at which binding is inhibited by 50%, determined using Spike proteins from WA1 and Beta variants. (**a**,**b**) show the binding inhibition activity (ID_50_) against WA1 and Beta and variants, respectively, for the three groups at three time points. Analysis of ID_50_ and the ELISA titer of the WA1 variant showed strong correlation between ID_50_ and the ELISA titer against full-length Spike (**c**) as well as the ELISA titer against RBD (**d**). Similar positive correlations were observed when analyses were carried out using data from all three time points and all three groups combined, as well as for individual groups. Error bars represent the geometric mean with a 95% CI. Statistical differences between groups were measured using a Kruskal–Wallis one-way ANOVA followed by a Dunn’s multiple comparison test. Asterisks indicate signifcant *p*-values: * *p* < 0.05; **** *p* < 0.0001.

The ability of immune sera to block the binding of Spike to the ACE2 receptor was evaluated by competitive ELISA. Receptor binding inhibition activity is expressed in terms of ID_50_, the serum dilution that inhibits binding of Spike to ACE2 by 50% (Figure 3a,b). Binding inhibition activity was evaluated using sera from three time points: two weeks post vaccination 2 (day 42), two weeks post vaccination 3 (day 126), and the last time point during the study (day 280). Assays were carried out using Spike proteins from two different variants of SARS-CoV-2: WA1 and Beta. Day 42 sera from the two conjugate groups showed strong inhibitory activity against WA1 spike with similar ID_50_ (Figure 3a). ID_50_ values for both conjugate groups were higher, but not significantly, than the mRNA group. On day 126, two weeks post vaccination 3, a similar pattern was observed, with no difference between the conjugate groups but significantly higher ID_50_ compared to the mRNA groups. By day 280, ID_50_ had declined for all three groups, consistent with the decrease in the antibody titer following the peak titers post dose 3. At this time point, all groups had a similar ID_50_.

A similar pattern was observed for binding inhibition activity against the Spike protein from the Beta variant at the three time points (Figure 3b). Here also, both conjugates had similar ID_50_s at all three time points. mRNA had a significantly lower ID_50_ compared to both conjugates on day 126 and compared to Conj-40 on day 42. The ID_50_s were similar for all three groups on day 280.

A strong correlation was observed between the ID_50_ and ELISA titer against the WA1 Spike (Figure 3c) when data combined from all three groups and all three time points were analyzed (Spearman r = 0.8004; *p* < 0.0001). Similarly strong correlations were also observed when the three groups were analyzed separately (Figure 3c). ID_50_ vs. the ELISA titer against RBD also yielded a strong correlation when all three groups were analyzed together (Spearman r = 0.6420; *p* < 0.0001), as well as moderate correlations when each group was analyzed separately (Figure 3d).

### 3.3. Virus Neutralization Activity of Immune Sera

The virus neutralization activity of sera from vaccinated animals was assessed using human OC43 coronaviruses expressing eGFP and spike proteins from SARS-CoV-2 WA1, Delta/B.1.617.2, Omicron/B.1.1.529, Omicron/EG.5.1, or Omicron/BA.2.86 variants [37]. The ability of immune sera to block infection of ACE2-expressing cells by viruses, measured by the reduction in the fraction of GFP-expressing cells, was used as a surrogate assay to evaluate its virus neutralizing activity. The ACE2-expressing BHK-21 cells were incubated with virus variants mixed with immune sera from each animal at different dilutions. ID_50_, defined as the serum dilution at which the fraction of cells expressing GFP is reduced by 50%, was determined for each animal serum. Geometric mean ID_50_ values for each group against different variants and at three time points are listed in Supplementary Table S1, and fold changes in ID_50_ values between different time points are listed in Supplementary Table S2. The conjugates, as well as the mRNA vaccine, in this study are based on the WA1 RBD and Spike protein, respectively. The virus neutralization activity of immune sera was lower against other variants. Supplementary Table S3 lists the fold changes in virus neutralization activity against Delta, B.1.1.529, BA2.86, and EG5.1 compared to WA1 activity at different time points: days 42, 126, and 280.

**Figure 4:**
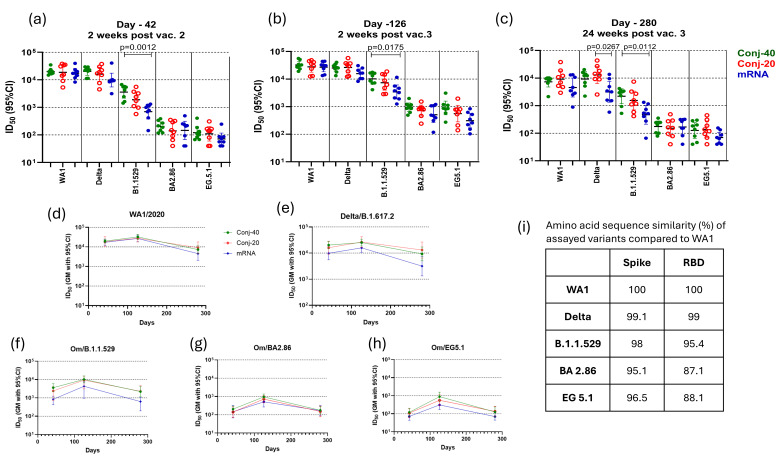
**Immune sera show strong virus neutralization activity:** Immune sera from animals vaccinated with RBD conjugate and mRNA were evaluated for virus neutralization activity using pseudo-typed OC43 Co-V expressing Spike protein from WT, Delta, Om/B.1.1.529, Om/BA2.86, and Om/EG5.1 variants of SARS-CoV-2. ID_50_, defined as the serum dilution that results in 50% inhibition of viral entry, was determined for each animal serum against different variants on day 42 (**a**), day 126 (**b**), and day 280 (**c**). All three groups showed similarly strong virus neutralization against WT and Delta variants but reduced neutralization activity against Omicron variants. (**d**–**h**) show the persistence of virus neutralization activity in the immune sera from animals vaccinated with Conj-40, Conj-20, and mRNA assayed against WT, Delta, Om/B.1.1.529, Om/B2.86, and Om/EG5.1 variants, respectively, during the study. (**i**) shows the sequence similarity of Spike and RBD of different variants compared to WA1. Error bars represent the geometric mean with a 95% CI. Statistical differences in ID_50_ between groups were measured using a Kruskal–Wallis one-way ANOVA followed by a Dunn’s multiple comparison test and are listed in Supplementary Table S3.

Figure 4a shows the ID_50_ of day 42 sera for each animal from the conjugate and mRNA groups, measured with different variants. All three groups produced a high ID_50_ (>17,000 GM titer) against the WA1 virus, indicating strong virus neutralizing activity against this variant. Against the Delta variant, the ID_50_ of the two conjugates was similarly high as against WA1. The mRNA group had a lower ID_50_ compared to the conjugate groups, but these differences were not statistically significant. The ID_50_ of all three groups was substantially lower against Omicron/B.1.1.529, and the mRNA group had a significantly lower ID_50_ compared to the Conj-40 group. Further reductions in ID_50_ were observed against the Omicron/BA2.86 and Omicron/EG5.1 variants. Overall, an approximately 2-log decrease in ID_50_ was observed for all three groups against BA2.86 and EG5.1 compared to WA1. Against Omicron EG 5.1 and BA 2.86, the ID_50_ of Conj-40 and Conj-20 was similar, and neither was significantly different from the mRNA group. The ID_50_ values for each group are listed in Supplementary Table S1, and the fold changes in ID_50_ against different variants compared to WA1 are listed in Supplementary Table S2. The amino acid sequence similarity of Spike and RBD of the different variants used in the virus neutralization assays compared to WA1 are listed in Figure 4i, and the amino acid sequence alignment is shown in Supplementary Figure S2.

A third vaccination, 12 weeks post vaccination 2, increased the virus neutralization activity of all three vaccine groups against all five variants (Figure 4b). Virus neutralization titers on day 126 were 1.6-, 1.5-, and 1.6-fold higher for Conj-40, Conj-20, and mRNA, respectively, against the WA1 virus compared to day 42, though this increase was not statistically significant (Supplementary Table S3). Similarly, a 1.3-, 1.7-, and 1.6-fold increase was observed for the respective three groups against the Delta variant compared to their day 42 values. All three groups showed a significantly higher ID_50_ on day 126 compared to day 42 against Omicron variants B.1.1.529, BA2.86, and EG5.1. For the B.1.1.529 variant, the increase was 2.8-, 3.9-, and 4.9-fold, respectively, for the three groups. The increase in ID_50_ from day 42 to day 126 was 4.7-, 5.2-, and 3.5-fold for BA2.86 and 7.4-, 5.1-, and 4.3-fold for EG5.1. Nevertheless, the ID_50_ of the three groups against Omicron variants was substantially lower than the day 126 values of the respective groups against WA1 and Delta (Figure 4b; Supplementary Table S1). As on day 42, the mRNA group showed a significantly lower ID_50_, compared to the Conj-40 group, against the B.1.1.529 variant (Figure 4b). The ID_50_ against this variant was 3.2- and 3.8-fold lower for the Conj-40 and Conj-20 groups compared to their ID_50_ against WA1, whereas the ID_50_ for the mRNA group decreased by 7.9-fold compared to WA1 (Supplementary Table S2). The ID_50_ of all three groups was further reduced against the Omicron EG 5.1 and BA 2.86 variants. The fold changes in the ID_50_ against different variants compared to WA1 at three time points for all three groups are listed in Supplementary Table S2.

On day 280, 24 weeks post vaccination 3, all three groups showed a significant reduction in virus neutralization activity against all five variants compared to the peak values on day 126 (Supplementary Tables S1 and S3). Nevertheless, the two conjugate groups retained a significant ID_50_ against WA1 and Delta that ranged between 7341 and 13,036 (GM titer) (Supplementary Table S1; Figure 4c–e). The ID_50_s for the mRNA group were lower than the conjugate groups against these two variants, but the difference was only significant against the Delta variant and when compared to the Conj-20 group (Figure 4c). While the virus neutralization ID_50_ on day 280 decreased from day 126 levels by 4.44- and 3.05-fold for the Conj-40 and Conj-20 groups, respectively, against the WA1 virus, the corresponding decrease for the mRNA group was 5.95-fold (Supplementary Table S3). Similarly, against the Delta variant, the virus neutralization ID_50_ decreased by 2.72- and 2.02-fold for the Conj-40 and Conj-20 groups, respectively, while the mRNA group decreased by 5.01-fold. As observed for day 42 and day 126 sera, the ID_50_ was reduced for all groups against the B.1.1.529 variant (Figure 4c; Supplementary Table S1): the ID_50_ for the mRNA group decreased by 7.30-fold compared to a 4.64- and 4.79-fold decrease for the Conj-40 and Conj-20 groups (Supplementary Table S3). The ID_50_ of the two conjugates was not significantly different against this variant, whereas the mRNA group was significantly lower when compared to the Conj-40 µg group (Figure 4c). The ID_50_ of all three groups was further reduced when assayed against the BA2.86 and EG5.1 variants, and all three groups produced a similar ID_50_ against these variants. Figure 4d–h show the profile of virus neutralization activity against different variants for the three groups on days 42, 126, and 280. The decrease from the peak virus neutralization activity on day 126 to day 280 was significant for all groups, except for Conj-20 against the Delta variant (Supplementary Table S3). The virus neutralization activity was relatively low for all three groups against the BA2.86 and EG5.1 variants on day 126. This decreased by 3- to 7-fold for different groups from day 126 to day 280 (Supplementary Table S3). When compared to the WA1 variant, the virus neutralization ID_50_ against these variants was substantially lower (34-to 240-fold lower) for all three groups at different time points (Supplementary Table S2).

**Figure 5:**
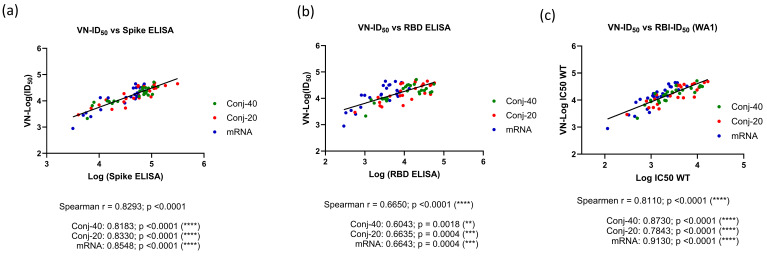
**Correlations were observed between virus neutralization activity and antibody titer**: Correlation between virus neutralization (VN) activity (ID_50_) and ELISA titer against WT full-length Spike (**a**) and RBD (**b**) were analyzed with data from all three groups and three time points (days 42, 126, and 280) combined, as well as with individual vaccine groups. Very strong or strong positive correlations were observed between the virus neutralization activity (VN-ID_50_) and ELISA titer against full-length Spike and RBD, respectively, when all groups were analyzed together, as well as individually. (**c**) shows the correlation between VN-ID_50_ and receptor binding inhibition (RBI-ID_50_) activity observed using WT Spike in the pseudo-virus and receptor binding inhibition assays. Very strong or strong correlations were observed when all three groups were analyzed together, as well as individually. Asterisks indicate significant *p*-values: ** *p* < 0.01; *** *p* < 0.001; **** *p* < 0.0001.

The virus neutralization ID_50_ from days 42, 126, and 280 against the WA1 virus showed a very strong correlation to the Spike ELISA titer (Spearman r = 0.8293; *p* = <0.0001) when all three groups were analyzed together (Figure 5a). An equally strong correlation was observed when individual groups were analyzed, yielding a Spearman r of 0.8183 (*p* < 0.0001), 0.8330 (*p* < 0.0001), and 0.8548 (*p* < 0.0001), respectively, for the Conj-40, Conj-20, and mRNA groups. The virus neutralization ID_50_ showed a strong correlation with the RBD ELISA titer, with a Spearman r = 0.6650 (*p* < 0.0001) when all three groups were analyzed together. Individual groups also showed a strong correlation, with a Spearman r = 0.6043 (*p* = 0.0018), 0.6635 (*p* = 0.0004), and 0.6643 (*p* = 0.0004), respectively, for the Con-40, Conj-20, and mRNA groups (Figure 5a,b). A similar analysis of the virus neutralization to ACE2 receptor binding inhibition activity of immune sera (ID_50_) also yielded a very strong correlation (Figure 5c). When all three groups were analyzed together, the Spearman r was 0.8110 (*p* = <0.0001). When the three groups were analyzed separately, Spearman r values of 0.8730 (*p* = <0.0001), 0.7843 (*p* = <0.0001), and 0.9130 (*p* = <0.0001) were observed against Conj-40, Conj-20, and mRNA, respectively (Figure 5c).

### 3.4. IgG Subclass Analyses

The IgG subclass distribution in the immune sera can have a significant effect on antibody-mediated functional activity. The serum levels of IgG1, IgG2, IgG1+3, IgG4, and IgM against WA1 Spike protein were determined for each animal on days 42, 126, and 280 for all three groups. Since the IgG3-specific secondary antibody is not available for rhesus macaques, a secondary antibody that reacts with both IgG1 and IgG3 was used for estimating IgG3 levels. Thus, the level of IgG3 could not be determined independently.

**Figure 6:**
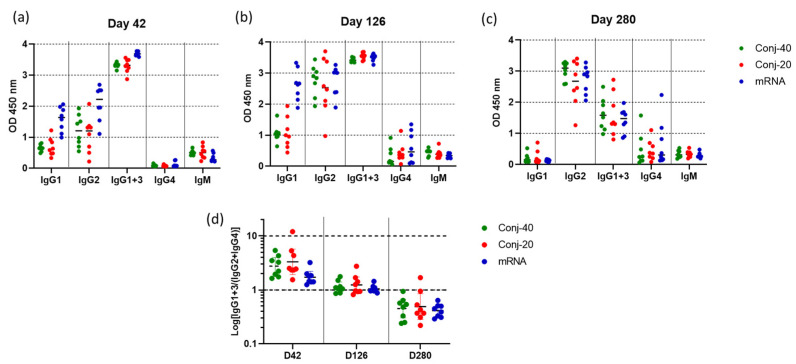
**Vaccinations with RBD conjugate and mRNA induce similar IgG subclass profiles:** IgG subclass levels in immune sera collected on (**a**) day 42 (2 weeks post vac 2), (**b**) day 126 (2 weeks post vac 3), and (**c**) day 280 (24 weeks post vac 3) from animals vaccinated with Conj-40, Conj-20, and mRNA. Error bars represent the geometric mean with a 95% CI. Since rhesus secondary antibody specific to IgG3 is unavailable, a secondary antibody that binds both IgG1 and IgG3 was used to estimate IgG1+3 levels. (**d**) shows the ratio IgG1+3/(IgG2+IgG4) for each group that shows a similar transition from a Th1-dominant (IgG1 and IgG3) subclass profile on day 42 to a Th2-dominant (IgG2 and IgG4) profile on day 280, with a balanced Th1/Th2 profile on day 126 for all three groups.

Figure 6a shows the levels of various IgG subclasses on day 42 (2 weeks post vaccination 2) sera for all three groups. IgG1 levels were low for all three groups. Nevertheless, the mRNA group had a significantly higher level of IgG1 than the conjugate groups (Figure 6 and Figure S3a). The two conjugate groups had similar levels of IgG1. IgG2 levels were also similar for the two conjugate groups but were higher for mRNA. While high levels of IgG1+3 were observed for all three groups, IgG4 levels were low. There were measurable levels of IgM for all three groups. Substantial changes in the IgG subclass distribution were observed in sera following the third vaccination. On day 126, two weeks post vaccination 3, IgG1 levels increased for the mRNA group compared to day 42, whereas the increase was marginal for the two conjugate groups (Figure 6b). IgG2 levels increased for all three groups from day 42 levels, and all three groups had similar levels of IgG2 on day 126. Similarly, IgG4 also increased from day 42 levels for all three groups, resulting in similar levels on day 126. IgM levels did not change from day 42 levels.

Further changes were observed in the IgG subclass profile on day 280, 24 weeks post vaccination 3 (Figure 6c). The level of IgG1 decreased to near zero for all three groups. IgG2 levels remained elevated for all the groups, at the same levels seen on day 126. IgG1+3 decreased significantly from day 126 levels, though all three groups produced similar IgG1+3 (Figure 6c and Figure S3c) on day 280. IgG4 also remained elevated at the day 126 levels for all three groups. All groups had low levels of IgM on day 280, similar to days 42 and 126.

**Figure 7:**
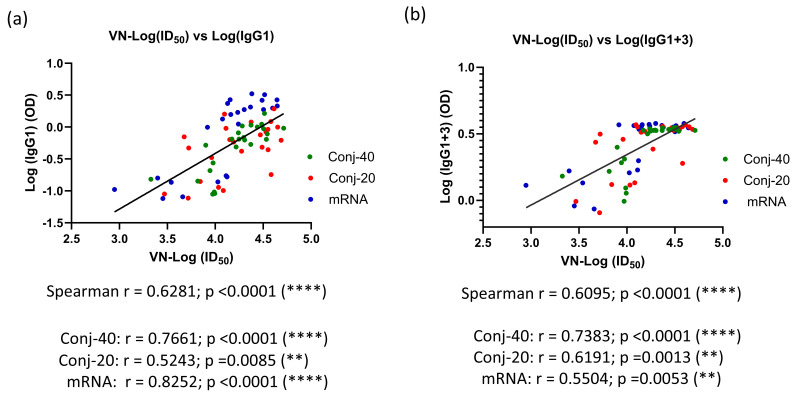
**Virus neutralization activity is strongly correlated with levels of IgG1 and IgG1+3 in immune sera:** Correlation between VN activity and levels of IgG subclass were analyzed for all three groups using data from days 42, 126, and 280. A strong correlation was observed between VN-ID_50_ and IgG1 levels in the immune sera (**a**). A similarly strong correlation was observed between VN-ID_50_ and IgG1+3 levels in the sera (**b**). Asterisks indicate significant *p*-values: ** *p* < 0.01; **** *p* < 0.0001.

Figure 6d shows the ratio of IgG1+3/(IgG2+IgG4) on days 42, 126, and 280 for the three groups. On day 42, all three groups had a ratio greater than 1, indicating a response dominated by cytophilic antibody. On day 126, this ratio is around 1, indicating a mixed response, whereas on day 280, the response has switched to one that is dominated by non-cytophilic antibodies, IgG2 and IgG4. During this period, the virus neutralization activity against the WA1 variant decreased significantly for all three groups. A correlation analysis was carried out between virus neutralization activity (WA1) and IgG subclass levels, including data from all three groups on days 42, 126, and 280. We observed strong positive correlations between the virus neutralization activity and IgG1, as well as IgG1+3 (Figure 7a,b), with Spearman r values of 0.6281 (*p* < 0.0001) and 0.6095 (*p* < 0.0001), respectively. On the contrary, IgG2 and IgG4 demonstrated no significant correlations, with Spearman r values of −0.0262 (*p* = 0.827) and −0.0554 (*p* = 0.6940), respectively.

### 3.5. RBD-EcoCRM Conjugate and mRNA/LNP Induce Antibodies with Similar Avidity

**Figure 8:**
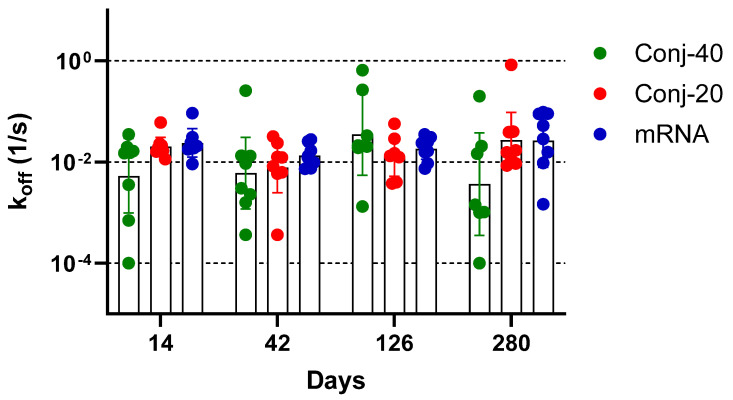
**Vaccination with RBD conjugate and mRNA induces antibodies with similar avidity:** Dissociation rate constant (k_off_) of immune sera collected on days 14, 42, 126, and 280 from animals vaccinated with Conj-40, Conj-20, and mRNA/LNP, determined by Biolayer Interferometry (BLI). Statistical analysis showed no significant difference in k_off_ between the three groups. No significant differences could be observed when data from different days were compared.

Avidity measurements have been used to assess the quality of the antibody generated by vaccination or by an infectious agent, with the goal of correlating it with functional activity. The binding affinity of an antibody, which may be correlated to its functional activity, is determined from the ratio of the association and dissociation rate constants of the interaction between the antibody and its target antigen. The determination of binding affinity requires knowledge of the concentration of the antigen-specific antibody in the immune sera, which may be challenging when the study involves a large number of serum samples. Nevertheless, determination of the dissociation constant, k_off_, does not require knowledge of the antigen-specific antibody concentration and can be carried out with immune sera. Several studies have employed Surface Plasmon Resonance or Biolayer Interferometry to measure the dissociation rate constant of immune sera as a measure of the antibody avidity of polyclonal sera [39,40,41,42,43,44]. We measured the dissociation rate constants of sera collected on days 14, 42, 126, and 280 from each animal vaccinated with Conj-40, Conj-20, and mRNA (Figure 8). All three groups generated antibodies with a similar avidity, with no significant differences in k_off_ between the groups on any given day or for the same groups between different days, indicating that the RBD conjugates in the AS01 adjuvant induce antibodies with a similar avidity as the mRNA-generated antibodies. This contrasts with our earlier observation with a malaria antigen, Pfs230D1, where we saw a decreasing trend in k_off_ (increase in avidity) with the increase in the number of vaccinations [40].

## 4. Discussion

SARS-CoV-2 Spike mRNA vaccines developed during the early days of the pandemic were crucial in controlling the pandemic. This rapid success was made possible due to the earlier work on SARS-CoV and MERS-CoV that identified the Spike as a target antigen [45,46,47], as well as the development of mRNA over recent decades as a feasible and rapidly deployed vaccine platform for humans [48,49,50,51,52,53]. Despite the historic speed developing and authorizing SARS-CoV-2 Spike mRNA vaccines, much of the globe remained without access to these highly efficacious tools until late in the emergency. In this rhesus macaque vaccination study, we showed RBD-EcoCRM, a vaccine that incorporates RBD antigen in a protein–protein radial conjugate formulated in a liposomal adjuvant, induces serum antibody levels and virus neutralization activity similar to or greater than that induced by full-length Spike in an mRNA/LNP vaccine (Pfizer/BioNTech) approved for human use.

Previous studies using SARS-CoV and MERS-CoV have demonstrated that vaccination with Spike antigen can induce a neutralizing antibody response against the virus in humans [45], and stabilizing it in a prefusion conformation may render it a better immunogen [47]. Adapting this to SARS-CoV-2, by two proline substitutions at positions 986 and 987 of the Spike protein, to stabilize the prefusion conformation has been critical to developing the successful vaccines based on this antigen [54]. Vaccination with full-length Spike antigen generates both neutralizing and non-neutralizing antibodies, and most neutralizing antibodies target the RBD of Spike [28]. Thus, the RBD antigen is an attractive option, as it can focus the immune response on the Spike binding site and reduce the non-neutralizing antibody generation. RBD-based vaccines have shown strong efficacy against COVID-19 in clinical trials and have been approved for human use [33,34,35].

We previously observed strong antibody responses, as well as virus neutralization activity, from RBD-EcoCRM conjugate with AS01 adjuvant in mouse immunogenicity studies [25]. A similar conjugate, RBD conjugated to Tetanus Toxoid, has been shown to have high efficacy against COVID-19 and was approved for human use in Cuba [33]. Additionally, RBD in an Alhydrogel adjuvant was shown to be efficacious and approved for human use in India [34]. These observations demonstrate that a full-length Spike may not be required to achieve efficacy, and smaller domains that are easier to produce may serve as equally effective vaccine antigens. Nevertheless, very few studies have directly compared the immune response generated by Spike versus RBD vaccines. Early preclinical and clinical studies of RBD and Spike on mRNA platforms by BioNTech showed very similar immunogenicity and virus neutralization activity for the two candidates [31,55].

RBD-EcoCRM conjugates in both 40 and 20 µg doses (Conj-40 and Conj-20, respectively) showed a strong antibody response against RBD, as well as WA1 Spike protein. These two antigen doses correspond to safe and effective doses of a similar protein conjugate vaccine against malaria, currently undergoing clinical trials. Though we did not see significant differences between the geometric mean of these two groups, anti-RBD titers of both groups were significantly higher than that generated by the mRNA vaccine. The antibody response against the full-length Spike protein was substantially higher than the response against RBD for the mRNA group, as expected, since the mRNA vaccine expresses full-length Spike antigen. Surprisingly, the RBD conjugate groups also showed an increased antibody response against Spike protein. This may indicate that the RBD antigen reveals additional epitopes that were cryptic in the Spike antigen, but the antibody generated against those bind efficiently to the Spike protein. Whether they contribute to functional activity is unclear. It is also possible that the adjuvanted RBD conjugate induces a more effective T-cell response, though T-cell responses were not evaluated in this study.

RBD conjugate and Spike mRNA vaccines are based on the WA1 antigen variant (WA1). Immune sera from all three groups demonstrated strong ACE2 receptor binding inhibition activity against WA1 and Beta variants post vaccinations 2 and 3, although inhibition activity was reduced against the Beta compared to the WA1 variant. For all three groups, activity waned by day 280. Conjugate groups, either high dose (day 42) or both (day 126), retained significantly higher binding inhibition activity compared to mRNA, but not on day 280. Binding inhibition activity measured against WA1 Spike was highly correlated with the antibody levels against Spike, as well as RBD.

The two-dose primary series of mRNA vaccines based on the WA1 Spike antigen approved for human use against SARS-CoV-2 was highly effective against severe disease [1,2]. But due to the waning of serum neutralizing antibody levels, a third dose was recommended to maintain high neutralizing antibody levels to protect from the various emerging variants of the virus such as Beta, Gamma, and Delta [56]. Even with a third dose, the virus neutralization activity of the vaccines was diminished against the highly mutated Omicron variants [57,58,59,60]. A third dose of Pfizer/BioNTech mRNA vaccine showed virus neutralization activity against Omicron B.1.1.529, even though the activity against this variant was lower by 8.3-fold compared to WA1 virus (virus neutralization titer of 891.4 vs. 107.6 against WA1 vs. B.1.1.529, respectively) [57]. Consistent with this result, the mRNA group in our rhesus study showed a 7.9- and 9.7-fold decrease in virus neutralization activity against Omicron B.1.1.529 compared to the WA1 virus at 2 and 24 weeks post vaccination 3, respectively (Supplementary Table S2). In comparison, for the Conj-40 group, virus neutralization activity against Omicron B.1.1.529 at the respective time points was 3.2- and 3.3-fold lower than that against the WA1 virus (Supplementary Table S2). Although all three groups showed high virus neutralization activity on day 126 against WA1, as well as the Delta variant, this activity significantly waned by day 280 (Supplementary Tables S1 and S3). This decrease was slightly greater for the mRNA group than the conjugate groups. Virus neutralization activity against the Omicron variants BA2.86 and Om EG5.1 was substantially lower compared to WA1 (Supplementary Tables S1 and S2).

In vaccinated and infected humans, Spike binding and neutralizing antibody levels are considered correlates of protection against severe disease caused by SARS-CoV-2 [61,62,63]. In this study, the virus neutralization activity of the conjugates and mRNA were highly correlated with the antibody titer against RBD, as well as Spike (Figure 5a,b). A strong correlation was also observed between the virus neutralization activity and receptor binding inhibition activity (Figure 5c).

mRNA vaccines are designed to generate intracellular antigen and induce an immune response primarily by MHC-I presentation of the peptide antigens. Conjugate, on the other hand, is extracellular and is expected to be internalized and processed for MHC-II presentation [64]. Although their mechanism of antigen presentation differs, RBD conjugates and mRNA groups induced a similar IgG subclass profile (Figure 6 and Figure S3). Additionally, changes in the IgG subclass distribution during the study were also very similar for the conjugate and mRNA groups. The IgG subclass profile induced by protein subunit vaccines can be influenced by the adjuvants used [65]. AS01 used to formulate the RBD conjugate has two immunostimulatory molecules, the TLR4 agonist MPL and the saponin-based adjuvant QS21 [66], and is approved for use in the Mosquirix^®^ [67] and SHINGRIX^®^ [68,69] vaccines. AS01 has been shown to induce Th1-biased immune responses [70].

In this study, the IgG subclass profile changed substantially from day 42 to day 280 (Figure 6a–d). On Day 42, two weeks post vaccination 2, IgG1+3 levels were high for all three groups, while IgG1 and IgG2 levels were moderate, IgG4 levels were near zero, and IgM levels were low. The third vaccination resulted in increases in IgG2 and IgG4, a moderate increase in IgG1, and continued high values of IgG1+3 on day 126. By day 280, following the 3rd vaccination, IgG2 levels were the highest, IgG1+3 levels had decreased, and IgG1 levels were near zero. The subclass distribution profiles generally remained similar among all three groups, although the mRNA group on days 42 and 126 had significantly higher IgG1 compared to the conjugate groups (Supplementary Figure S3). IgG2 and IgG4 levels have been observed to increase in humans receiving multiple administrations of mRNA vaccine [71,72,73,74]. In a rhesus study, Routhu et. al. compared the immune response from Novavax’s Matrix-M adjuvanted protein vaccine (NVX-CoV2373) with Moderna’s mRNA vaccine (mRNA-1273), both having WA1 Spike as the antigen [75]. Two weeks post the second vaccination, both groups showed an IgG subclass profile dominated by IgG1. The third vaccination resulted in a substantial decrease in IgG1 levels, with a concomitant increase in IgG4, while IgG2 and IgG3 levels were unchanged. A similar effect was observed in our previous NHP study of a malaria conjugate vaccine with ALFQ adjuvant, a liposomal adjuvant with similar components as AS01 [40].

While all four IgG subclasses can inhibit viral entry into cells, cytophilic antibodies IgG1 and IgG3 are efficient in facilitating Fc-mediated activities, such as antibody-dependent cellular cytotoxicity (ADCC), antibody-dependent cellular phagocytosis (ADCP), and complement-dependent cytotoxicity (CDC) that are important for viral clearance. Although vaccination induced an initial immune response dominated by cytophilic antibodies IgG1 and IgG3, the subclass profile switched to a subclass distribution dominated by IgG2 and IgG4 during the 24-week period following the third vaccination. During this period, virus neutralization activity against the WA1 virus also decreased significantly by 4.4-fold from peak activity on day 126 (Supplementary Table S3). Correlation analysis showed a strong positive correlation between virus neutralization activity and IgG1, as well as IgG1+3, levels (Figure 7), whereas no significant correlation was observed with IgG2 and IgG4 levels. This indicates the stronger virus neutralization potential of the cytophilic antibodies IgG1 and IgG3, and their natural waning and class switching following vaccination results in the loss of vaccine-induced protection. Both conjugates and mRNA showed very similar correlations between virus neutralization activity and IgG subclass levels, demonstrating the similarity in the immune response between the two vaccines. The loss of antiviral activity is further exacerbated by escape mutations due to ongoing viral evolution, necessitating updated vaccines targeting current variants [76].

mRNA vaccines have been extremely successful in controlling COVID-19, and mRNA has proven to be a technology platform for the rapid development of effective vaccines against infectious diseases. Nevertheless, the occurrence of rare side effects, such as myocarditis and allergic reactions, attributed to mRNA vaccines has resulted in some level of hesitancy in the general population toward mRNA vaccines [14,15,16,77]. Therefore, equally efficacious protein-based vaccine options against infectious agents like SARS-CoV-2 and other emerging viral pathogens are needed. Protein conjugates described here can be easily manufactured and combined with adjuvants that are safe and already approved for human use, as demonstrated by the success of the similar protein conjugate vaccine approved for use against SARS-CoV-2 [33], as well as the ongoing and completed clinical trials of malaria vaccines ([23,24], Clinical Trial ID: NCT03917654).

Successful cGMP manufacturing and clinical testing of protein–protein conjugates by us, as well as other groups, have demonstrated clearly that manufacturing and regulatory challenges are not significant barriers to vaccine development using this technology. Protein–protein conjugates synthesized by the same chemical conjugation technology have been shown to be safe in human studies [22,23,24]. Since the manufacturing process uses the well-established technologies of protein production and relatively simple chemical conjugation, manufacturing of the conjugate can be carried out anywhere with moderate manufacturing that exists even in low- and middle-income countries. This can facilitate easy access to effective vaccines worldwide, especially in an emergency. Additionally, the RBD-EcoCRM conjugate can be readily adapted to address viral evolution by substituting the receptor binding domain of the currently circulating variant or a combination of conjugates to generate a multivalent vaccine targeting multiple variants. One of the drawbacks of the mRNA vaccine has been the required cold storage at very low temperatures, making its transport and distribution very challenging, especially in tropical countries. A vaccine that is stable at moderate temperatures would be extremely valuable under these conditions. Additional studies need to be carried out to assess the long-term storage stability and temperature sensitivity of the RBD-EcoCRM conjugate, and the stability of protein conjugates in general.

Many of the successful COVID vaccines developed during the pandemic were designed to generate an antibody response against Spike or RBD. Higher levels of antibody response also resulted in higher levels of neutralizing antibody in the sera. A correlation between neutralizing antibody levels and vaccine efficacy was established from human studies [61,62,63]. In this study, we assessed the correlation between the virus neutralization activity (functional activity assay) and the total antibody response against the antigen, IgG subclass levels induced by vaccination and binding inhibition activity, a surrogate functional assay. In these analyses, we found a strong positive correlation between the virus neutralization activity and total antibody response (Figure 5a,b). We also observed a similarly strong positive correlation between the ACE2 receptor binding inhibition activity and total antibody titer (Figure 3c,d). These results show that the antibody titer and receptor binding inhibition activity may provide easy readouts for vaccine activity. We also saw a positive correlation between functional activity and levels of IgG1 and IgG1+3, revealing the contribution of these IgG subclasses to the functional activity. We analyzed individual groups to determine if any of the groups behave differently compared to others. Our analyses show a very similar dependence for each group in all these assays, further confirming the similarity of the immune response between mRNA and the RBD conjugate.

The RBD conjugate demonstrated strong immunogenicity in the NHP study described here. Antibody titers and neutralizing activity induced by the conjugate were similar or better than those induced by the licensed mRNA vaccine from Pfizer/BioNTech. Both the mRNA and conjugate induced very similar IgG subclass profiles on vaccination and exhibited similar class switching during the 6-month period after vaccination. The mRNA and RBD conjugate generated antibodies with similar avidity. Based on these observations, we conclude that the RBD conjugate with the AS01 adjuvant induces an immune response in rhesus that is qualitatively and quantitatively similar to the mRNA/LNP vaccine approved for human use.

This study had limitations. The mRNA used in this study was obtained from residual volumes in vials that had been reconstituted for human use; fresh vials of the vaccine were not available for other than human use at the time of this study. Unused mRNA collected from opened vials was frozen and kept at −80 °C until use. This process involved subjecting the mRNA/LNP formulation to an additional freeze–thaw cycle that was not recommended for vials used for human vaccination. This precautionary measure was recommended to avoid any potential impact on the vaccine potency by additional freeze–thaw cycles. Nevertheless, subsequent studies of Pfizer/BioNTech and Moderna mRNA/LNP vaccines have demonstrated that the refreezing of residual vaccine from opened vials, followed by long-term storage at −20 °C or −80 °C and thawing, have no adverse effect on the immunogenicity of these vaccines [78]. While there are no reported studies on the effect of multiple freeze–thaw cycles on mRNA/LNP, studies using siRNA/LNP have shown no loss of siRNA activity with three cycles of freeze–thaw when cryoprotectants such as sucrose or trehalose are present as an excipient in the formulation [79]. Pfizer/BioNTech and Moderna mRNA/LNP vaccines have sucrose as an excipient, which may provide protection during freeze–thaw cycles [80].

EcoCRM^®^ used in this study as a carrier for the RBD conjugate has not been tested in human clinical trials. It is currently being developed as a biosimilar of CRM197, a carrier used in polysaccharide vaccines [26,81]. However, our preclinical studies using two different malaria antigens comparing EcoCRM^®^ and CRM197 showed a very similar immune response induced by the two carriers [21]. EcoCRM^®^ is developed as a cheaper option to CRM197, suitable for vaccine development targeted to low- and middle-income countries.

## 5. Conclusions

We conclude that the rhesus macaque antibody responses induced by the RBD-EcoCRM conjugate and the mRNA vaccine are similar. Conjugate with the AS01 adjuvant induced similar or better antibody levels against RBD and full-length Spike after each vaccination and the approximate six months following the 3rd vaccination. The receptor binding inhibition and virus neutralization activities of the vaccines were also very similar and correlated with the antibody response. The virus neutralization activity diminished with the viral evolution for both vaccines. The IgG subclass profiles induced by vaccination also were very similar for the conjugate and mRNA following each vaccination and during the six months following the last vaccination. The IgG subclass profile switched from the initial cytophilic IgG1- and IgG3-dominated response after vaccination to an IgG2- and IgG4-dominated profile during the subsequent six-month period. This, along with the natural waning of the antibody levels, may explain the decline in virus neutralization activity over time after the 3rd vaccination. The antibody avidity of the polyclonal sera at different time points during the study also was similar between the conjugate and mRNA. These results demonstrate that the immune response induced in rhesus macaques by the RBD-EcoCRM conjugate adjuvanted with AS01 is not inferior to that induced by the highly efficacious mRNA vaccine approved for human use. This clearly shows the potential for the RBD-EcoCRM conjugate as a vaccine against SARS-CoV-2 and the protein–protein conjugate as a vaccine platform for future vaccine development.

## Data Availability

The datasets generated and/or analyzed during the current study are available from the corresponding author on reasonable request.

## Supplementary Materials

The following supporting information can be downloaded at: https://www.mdpi.com/article/10.3390/vaccines13060648/s1, Figure S1: Schematic representation of RBD-EcoCRM synthesis; Figure S2: Sequence alignment; Figure S3: Vaccinations with RBD conjugate and mRNA induce similar IgG subclass profiles; Table S1: VN-ID_50_ for three groups at three time points (days 42, 126, and 280) against different SARS-CoV-2 variants; Table S2: Fold increase (+) or decrease (−) in VN-ID_50_ against different variants compared to WA1, at various time points; Table S3: Fold increase (+) or decrease (−) in VN-ID_50_ between time points, against different variants.

## Abbreviations

The following abbreviations are used in this manuscript:

ACUC	Animal Care and Use Committee
BIA	Binding inhibition activity
BLI	Biolayer interferometry
Cys	Cysteine
ELISA	Enzyme-linked immunosorbent assay
GEE	General estimating equation
LMICs	Low- and middle-income countries
NHP	Nonhuman primate
NIAID	National Institute of Allergy and Infectious Diseases
RBD	Receptor binding domain
RT	Room temperature
OD	Optical density
VN	Virus neutralization
