# Supplementary material for: SARS-CoV-2 Receptor Binding Domain (RBD) Protein–Protein Conjugate Induces Similar or Better Antibody Responses as Spike mRNA in Rhesus Macaques
Source: Vaccines (Basel). 2025 Jun 17;13(6):648. doi: 10.3390/vaccines13060648 (PMC12197534; doi:10.3390/vaccines13060648)
Supplement: Supplementary file 1 [file vaccines-13-00648-s001.zip › vaccines-3672293-supplementary.pdf]

SARS-CoV-2 receptor binding domain (RBD) protein–protein conjugate induces similar or better antibody responses as Spike mRNA in rhesus macaques

Authors: Puthupparampil V Scaria, Christopher G. Rowe, Ivan Kosik, Zhe Hu, Jonathan P. Renn, Nada Alani, Pinar Kemanli, Sachy Orr-Gonzalez, Lynn Lambert, Kayode Adeyemi, Justin Y A Doritchamou, Emma K Barnafo, Kelly M Rausch, Liya Muslinkina, Robert D Morrison, John-Paul Todd, Dominic Esposito, Andrew Lees, Jonathan Yewdell, and Patrick E. Duffy

Supplementary Material

Supplementary Figures S1–S3

Supplementary Tables S1–S3

Supplemental Figure S1: Schematic representation of RBD-EcoCRM synthesis.

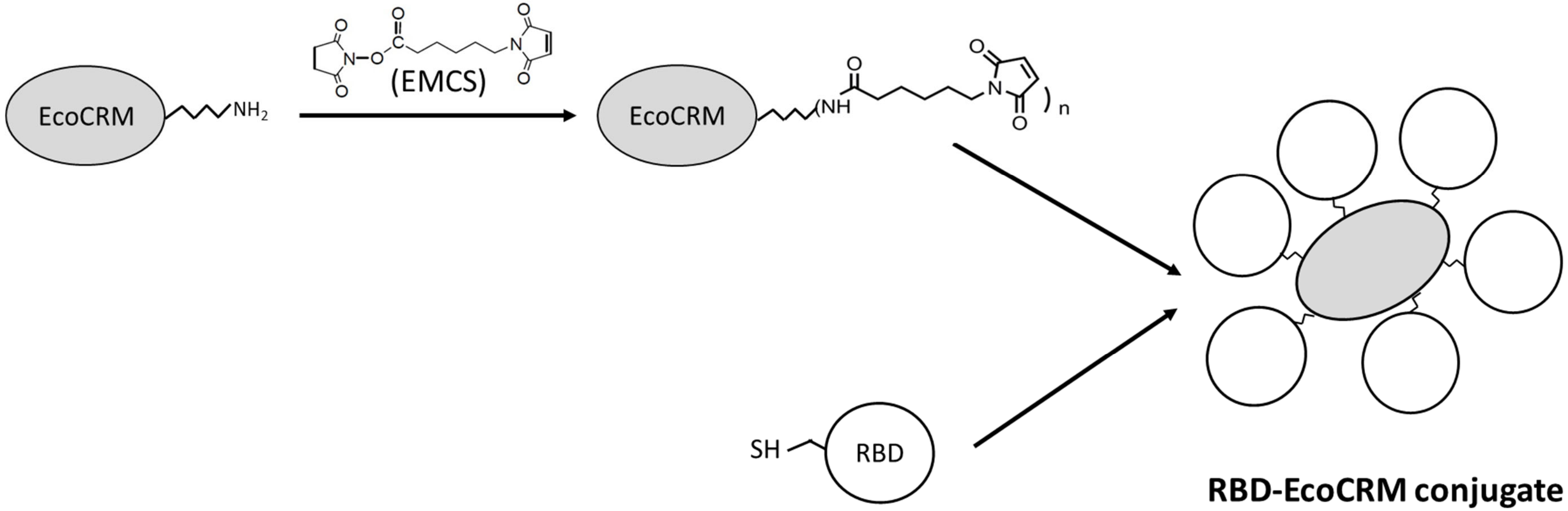

Supplemental Figure S2: Sequence alignment.

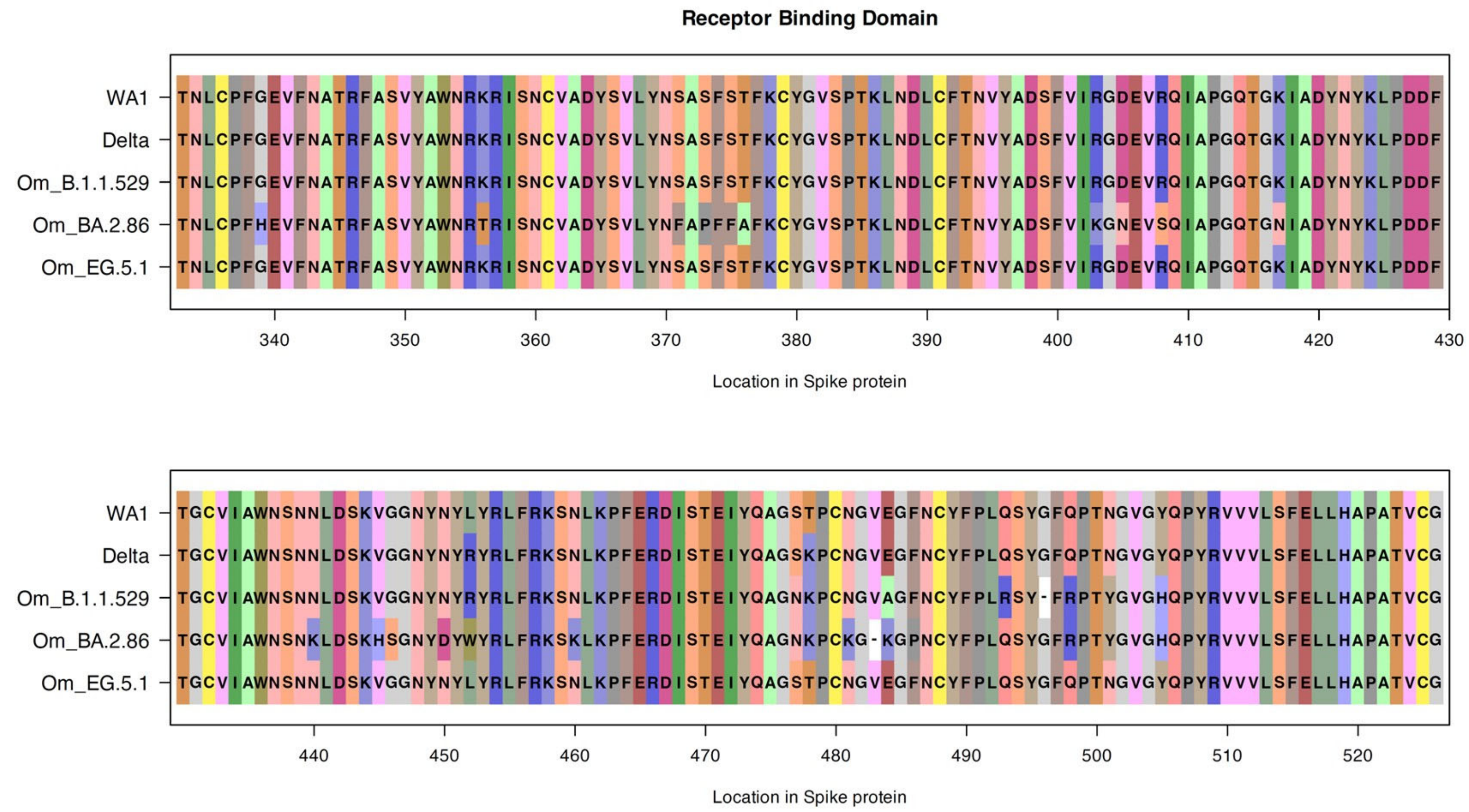

Supplemental Figure S3: Vaccinations with RBD conjugate and mRNA induce similar IgG subclass profiles: Levels of (a) IgG1, (b) IgG2, (c) IgG1+3, (d) IgG4, and (e) IgM induced by vaccination of Rhesus macaques with Conj-40, Conj-20, and mRNA in sera collected on D42 (2 weeks post vac 2), D126 (2 weeks post vac 3), and D280 (24 weeks post vac 3). Error bars represent the geometric mean with a 95% CI. Statistical

differences between groups were measured using a Kruskal–Wallis one-way ANOVA followed by a Dunn's multiple comparator test. * $p \leq 0.05$, ** $p \leq 0.01$, *** $p \leq 0.001$, **** $p \leq 0.0001$.

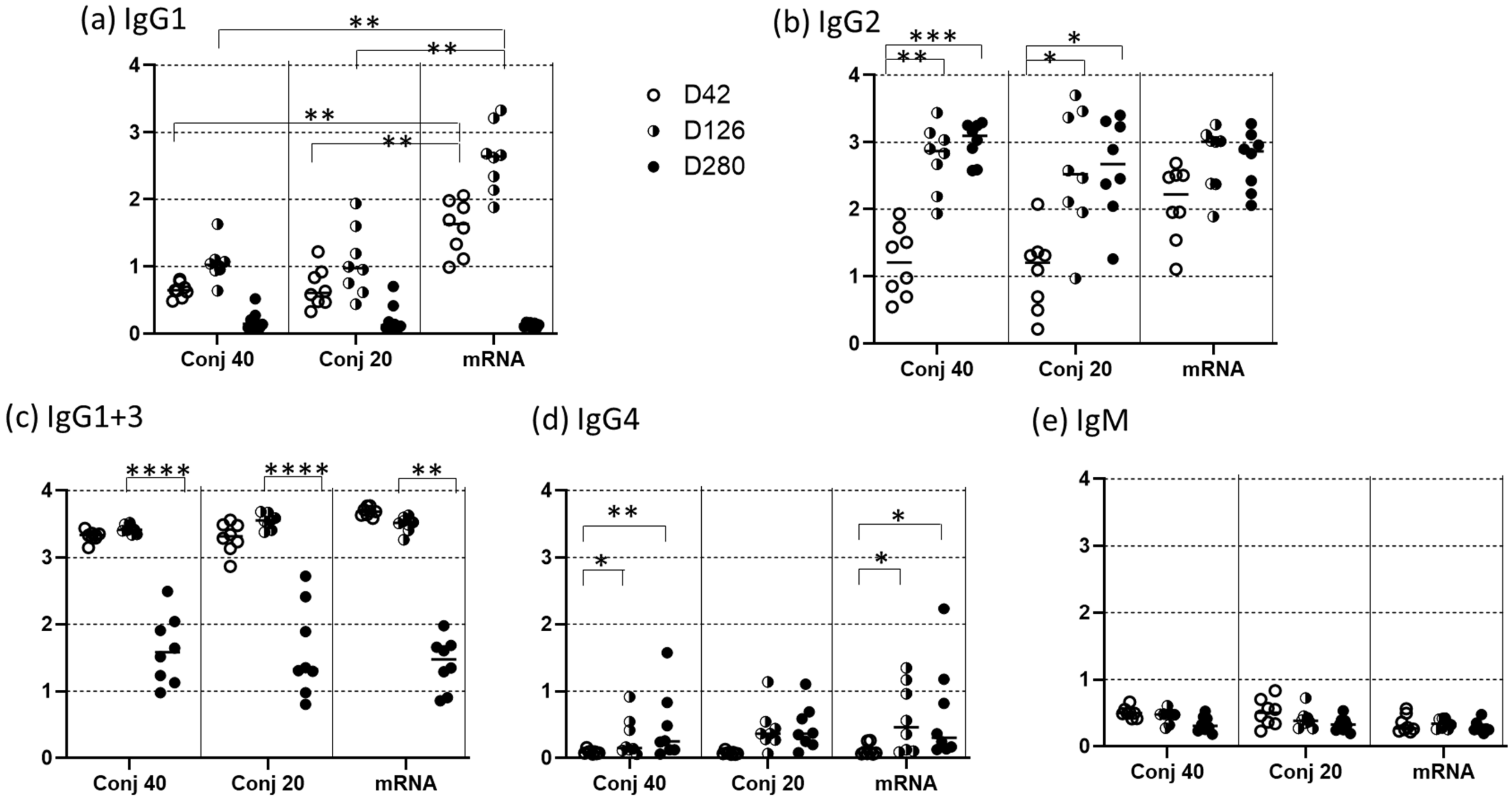

Supplementary Table S1. VN-ID$_{50}$ for three groups at three time points (days 42, 126, and 280) against different SARS-CoV-2 variants.

	**Conj-40**			**Conj-20**			**mRNA**		
	**d-42**	**d-126**	**d-280**	**d-42**	**d-126**	**d-280**	**d-42**	**d-126**	**d-280**
**WA1**	19922	32564	7341	18688	27827	9123	17285	27080	4555
**Delta**	20146	25183	9259	15981	26374	13036	9810	15830	3162
**B.1.1.529**	3603	10186	2195	1902	7406	1546	701	3416	468
**BA2.86**	204	953	176	140	724	152	146	512	169
**EG5.1**	121	896	126	110	562	135	72	311	70

Supplementary Table S2. Fold increase (+) or decrease (-) in VN-ID_{50} against different variants compared to WA1, at various time points.

	**Day**	**Conj-40**	**Conj-20**	**mRNA**
**Variant**		**WA1**	**WA1**	**WA1**
**WA1**	d-42	1	1	1
	d-126	1	1	1
	d-280	1	1	1
**Delta**	d-42	+1.01	-1.16	-1.76
	d-126	-1.29	-1.05	-1.71
	d-280	+1.26	+1.43	-1.44
**B.1.1.529**	d-42	-5.53	-9.82	-24.7
	d-126	-3.19	-3.75	-7.92
	d-280	-3.34	-5.90	-9.73
**BA2.86**	d-42	-97.6	-133	-118.3
	d-126	-34.2	-38.4	-52.9
	d-280	-41.7	-60.0	-26.9
**EG5.1**	d-42	-164.6	-169	-240.1
	d-126	-36.3	-49.5	-87.1
	d-280	-58.2	-67.6	-65.1

Supplementary Table S3. Fold increase (+) or decrease (-) in VN-ID_{50} between time points, against different variants.

		Conj-40		Conj-20		mRNA	
		**d-126**	**d-280**	**d-126**	**d-280**	**d-126**	**d-280**
**WA1**	**d-42**	+1.63 (ns)	-2.71 (*)	+1.50 (ns)	-2.05 (ns)	1.57 (ns)	-3.79 (*)
	**d-126**		- 4.4 (****)		-3.05 (*)		-5.95 (***)
**Delta**	**d-42**	+1.25 (ns)	-2.17 (ns)	+1.65 (ns)	-1.23 (ns)	1.61 (ns)	-3.1 (ns)
	**d-126**		-2.72 (**)		-2.02 (ns)		-5.01 (**)
**B.1.1.529**	**d-42**	+2.83 (*)	-1.64 (ns)	+3.89 (*)	-1.23 (ns)	+4.87 (**)	-1.49 (ns)
	**d-126**		-4.64 (***)		-4.79 (*)		-7.30 (***)
**BA2.86**	**d-42**	+4.67 (**)	-1.16 (ns)	+5.17 (*)	+1.08 (ns)	+3.50 (**)	+1.16 (ns)
	**d-126**		-5.41 (**)		-4.76 (*)		-3.03 (**)
**EG5.1**	**d-42**	+7.40 (**)	+1.04 (ns)	+5.12 (**)	+1.23 (ns)	+4.32 (*)	-1.03 (ns)
	**d-126**		-7.11 (***)		-4.16 (*)		-4.44 (*)

* $p \leq 0.05$, ** $p \leq 0.01$, *** $p \leq 0.001$, **** $p \leq 0.0001$
